# AI-Driven Real-Time Monitoring of Cardiovascular Conditions With Wearable Devices: Scoping Review

**DOI:** 10.2196/73846

**Published:** 2025-11-11

**Authors:** Ali Abedi, Anshul Verma, Dherya Jain, Jathushan Kaetheeswaran, Cynthia Chui, Milad Lankarany, Shehroz S Khan

**Affiliations:** 1 KITE Research Institute Toronto Rehabilitation Institute University Health Network Toronto, ON Canada; 2 Institute of Biomedical Engineering University of Toronto Toronto, ON Canada; 3 Krembil Brain Institute University Health Network Toronto, ON Canada; 4 Library & Information Services Toronto Rehabilitation Institute University Health Network Toronto, ON Canada; 5 College of Engineering and Technology American University of the Middle East Egaila Kuwait

**Keywords:** cardiovascular diseases, artificial intelligence, real-time monitoring, continuous monitoring, wearable devices, machine learning, deep learning, smartwatches, electrocardiography (ECG), atrial fibrillation, mobile phone

## Abstract

**Background:**

Cardiovascular diseases remain the leading cause of mortality worldwide, accounting for 18 million deaths annually. Detection and prediction of cardiovascular conditions are essential for timely intervention and improved patient outcomes. Wearable devices offer a promising, noninvasive solution for continuous monitoring of cardiovascular signals, vital signs, and physical activity. However, the large data volumes generated by these devices and the rapid fluctuations in cardiovascular signals necessitate advanced artificial intelligence (AI) techniques for real-time analysis and effective clinical decision-making.

**Objective:**

The objective of this scoping review was to identify the main challenges of AI-driven platforms for real-time cardiovascular condition monitoring with wearable devices and explore potential solutions. In addition, this review aimed to examine how AI algorithms are developed for robust monitoring and how deployment pipelines are optimized to enable real-time cardiovascular condition monitoring.

**Methods:**

A comprehensive search was conducted in the following electronic databases: MEDLINE(R) ALL (Ovid), Embase (Ovid), Cochrane Central Register of Controlled Trials (Ovid), Web of Science Core Collection (Clarivate), IEEE Xplore, and ACM Digital Library, yielding 2385 unique records. Inclusion criteria focused on studies that used wearable devices for participant data collection and applied AI algorithms for real-time analysis to detect or predict cardiovascular events and diseases. After title and abstract screening, 153 papers remained, and following a full-text review, 19 studies met the inclusion criteria.

**Results:**

The findings indicate that despite the promise of AI and wearable devices, research on real-time cardiovascular monitoring remains limited and lacks comprehensive validation. Most studies relied on publicly available wearable datasets rather than real-world validation with recruited participants in community settings. Studies that deployed AI algorithms in real time frequently failed to report operational characteristics and challenges. Electrocardiography-based wearable sensors were the most frequently used devices, primarily in hospital settings. A variety of AI techniques, ranging from traditional machine learning to lightweight deep learning algorithms, were deployed either on wearable devices or via cloud-based processing.

**Conclusions:**

Robust, interdisciplinary research is needed to harness the full potential of AI-driven, real-time cardiovascular health management using wearable devices. This includes the development and validation of scalable solutions for continuous community-based deployment. Furthermore, real-world challenges such as participant compliance, hardware and connectivity constraints, and AI model optimization for real-time continuous monitoring must be carefully addressed.

## Introduction

### Background

Cardiovascular diseases (CVDs) continue to be the leading cause of mortality worldwide, causing more than 18 million deaths each year [1]. The number of deaths attributed to CVDs has increased significantly over the past decades, rising by 53.7% from 12.1 million in 1990 to 18.6 million in 2019 [2]. This trend is expected to continue, with an estimated 35.6 million cardiovascular deaths by 2050, an increase of 90% compared with 2025 [2]. This escalating trend underscores the urgent need for comprehensive prevention and management strategies to address the global impact of CVDs.

Detection and prediction of CVDs and cardiovascular events are critical for reducing the morbidity and mortality associated with these conditions [3-5]. CVDs, such as coronary artery disease, hypertensive heart disease, and cardiomyopathies, often develop silently over years, with patients exhibiting minimal or no symptoms until a significant event occurs [6]. Identifying risks at an early stage allows for timely intervention, lifestyle modifications, and treatment, which can dramatically reduce the likelihood of severe outcomes [7]. Real-time detection and prediction of cardiovascular events, such as heart attacks, cardiac arrest, and strokes, is particularly essential because it provides actionable insights during critical windows when interventions can have the greatest impact. Delays in recognizing and responding to subtle physiological changes can mean the difference between prevention and a life-threatening event [8,9].

Wearable devices equipped with sensors for continuous monitoring of vital signs such as heart rate, blood pressure, and oxygen saturation offer the potential to identify abnormal patterns indicative of cardiovascular stress or disease [10]. These devices enable remote monitoring for people in the community, allowing health care providers to track patients’ health in real time and intervene promptly, even outside of clinical settings. Coupled with artificial intelligence (AI), these devices can analyze large amounts of data in real time, detecting and predicting potential cardiovascular events before they occur [9]. Advanced machine learning and deep learning techniques play a critical role in processing and interpreting the large and complex data captured by wearable devices, identifying subtle patterns and trends that may go unnoticed by human observation. Their ability to learn from historical data and continuously improve predictions ensures timely and accurate alerts, enabling health care providers to intervene before critical events unfold, thereby reducing the risk of adverse outcomes [9].

An AI-driven, wearable sensor-based platform for real-time cardiovascular monitoring can be equipped with single or multimodal sensors to collect various data, including electrocardiography (ECG), photoplethysmography (PPG), heart rate, blood pressure, and mobility indicators. These data are analyzed by AI algorithms, trained on historical datasets, and optimized for real-time inference. Depending on the system’s design, these algorithms can run either directly on wearable devices or on cloud servers (requiring data transfer). Analysis results are then communicated to clinicians, participants, or caregivers, enabling timely interventions to prevent or reduce adverse cardiovascular outcomes.

By prioritizing early detection and prediction, health care systems can shift their focus from reactive to proactive care, reducing hospitalizations, improving health outcomes and patients’ quality of life, and lowering health care costs [11]. Ultimately, early intervention is the foundation of effective CVD management and a pathway to saving millions of lives annually [3-5].

### Related Reviews

Table 1 outlines recent reviews on the applications of wearable devices and AI algorithms for cardiovascular monitoring [10,12-22], as well as relevant reviews in the broader domain of health care monitoring [23-25]. Each review is marked to indicate which of the 4 key concepts (CVD, real-time monitoring, AI, and wearable devices) it covers. While previous reviews on cardiovascular monitoring addressed 1 or 2 concepts, wearable devices [10,12-17,20-22], AI [18,20-22,25], or real-time monitoring [10,23-25], none comprehensively covered all 3. In addition, reviews on real-time monitoring in general health care [23-25] lack a specific focus on the detection and prediction of cardiovascular conditions. Most of these reviews included studies that used precollected public datasets for developing AI algorithms, which do not deal with the real-world challenges of deploying AI for real-time monitoring.

**Table 1:** A comparison between this scoping review and previous related reviews, sorted by the inclusion of focus on cardiovascular diseases, real time, artificial intelligence, and wearable concepts^a^.

Review	Year	CVD^b^	Real time	AI^c^	Wearable
Albahri et al [23]	2018	✗	√	✗	✗
Uddin and Koo [24]	2024	✗	√	✗	✗
Paganelli et al [25]	2022	✗	√	√	✗
Bogar et al [12]	2024	√	✗	✗	√
Ho et al [13]	2024	√	✗	✗	√
Duncker et al [14]	2021	√	✗	✗	√
Bayoumy et al [15]	2021	√	✗	✗	√
Nazarian et al [16]	2021	√	✗	✗	√
Scholte et al [17]	2024	√	✗	✗	√
Ahsan and Siddique [18]	2022	√	✗	√	✗
Safdar et al [19]	2018	√	✗	√	✗
Lee et al [20]	2022	√	✗	√	√
Huang et al [21]	2022	√	✗	√	√
Moshawrab et al [22]	2023	√	✗	√	√
Lin et al [10]	2021	√	√	✗	√
This review	2025	√	√	√	√

^a^This review is the only one that integrates all 4 concepts comprehensively.

^b^CVD: cardiovascular disease.

^c^AI: artificial intelligence.

### Research Questions

AI [18,20-22,25] plays a crucial role in processing data from wearable devices [10,12-17,20-22] facilitating real-time [10,23-25] detection, prediction, and diagnosis of cardiovascular conditions. This empowers clinicians to intervene swiftly, helping to prevent or mitigate adverse outcomes, reduce hospitalizations and mortality, and improve patient quality of life. In this paper, a scoping review was conducted to systematically map the current body of research on AI-driven platforms using wearable sensors for real-time cardiovascular monitoring and to identify knowledge gaps and associated challenges. The scoping review was guided by the following research questions: (1) What are the primary challenges of AI-driven platforms for real-time cardiovascular condition monitoring with wearable devices, and how are these challenges addressed? (2) How are AI algorithms developed to support robust cardiovascular condition monitoring? (3) How are AI algorithms and deployment pipelines optimized to enable real-time cardiovascular condition monitoring?

While the first research question focuses on platform-level challenges and solutions for real-time cardiovascular condition monitoring, the second and third questions specifically examine AI algorithms, one of the core components of these platforms, in terms of ensuring robustness and enabling real-time deployment.

## Methods

### Study Design

This study used a scoping review methodology to address the broad scope of the research questions, the diversity of the studies and populations, and the absence of previously conducted comprehensive reviews [26,27]. The scoping review followed the framework outlined by Arksey and O’Malley [26] and was reported in compliance with the PRISMA-ScR (Preferred Reporting Items for Systematic Reviews and Meta-Analyses extension for Scoping Reviews) checklist (Multimedia Appendix 1) [27].

### Eligibility Criteria

#### Inclusion Criteria

Peer-reviewed journal and conference papers written in English, including quantitative, qualitative, and mixed method studies, were considered for inclusion. To be eligible, studies had to describe either the development of a new platform (research-based or commercial) or the use of an existing platform for AI-driven real-time monitoring of cardiovascular conditions through wearable devices. The platform was required to meet all four criteria: (1) It must collect continuous data from adult participants using wearable sensing devices. (2) It must be AI-driven, incorporating machine learning and deep learning algorithms to analyze wearable data. (3) The AI algorithms must perform data analysis and inference-making in real time or near real time. (4) The inferences made by AI algorithms must involve the detection or prediction of cardiovascular conditions, diseases, or events. Studies could also look at other comorbidities, such as diabetes, in addition to CVD.

Real-time analysis involves continuous processing with minimal delay, while near–real time refers to intermittent or slightly delayed processing due to computational or design limitations. Although “detection” and “prediction” are often used interchangeably in the literature, this paper distinguishes between them: detection infers current conditions from current wearable data, whereas prediction uses current data to forecast future conditions.

Using the SPICE framework [28], the setting; population; intervention; comparison; and evaluation criteria were identified as participants’ homes, nursing homes, or hospitals; adult participants; monitoring (predicting or detecting) any cardiovascular condition; real-time AI algorithms; and the accuracy and latency of real-time AI algorithms, respectively.

#### Exclusion Criteria

Non–peer-reviewed and non-English publications or resources were excluded. Studies were excluded if they (1) did not use a platform in which wearable sensor devices were used for data collection from adult participants, (2) did not use AI algorithms for wearable sensor data analysis, (3) performed offline data analysis, or (4) evaluated their platform only on wearable sensor datasets already collected, such as publicly available datasets.

If 1 or more of the exclusion criteria were met, studies were excluded.

### Information Sources and Search Strategy

To identify relevant studies, a comprehensive literature search was designed in collaboration with an information specialist (CC) and further refined through team discussions. AA and AV initially provided CC with a list of keywords and 20 target papers that needed to be retrieved from the search. Following this, CC, AA, and AV collaboratively refined the keyword list and developed a search strategy using a combination of subject headings and text words for MEDLINE, which was subsequently translated to other databases. The search strategy consisted of 4 concepts: wearable devices, CVDs, AI algorithms, and real-time data analysis. The search was conducted in the following electronic bibliographic databases: MEDLINE(R) ALL (Ovid), Embase (Ovid), Cochrane Central Register of Controlled Trials (Ovid), Web of Science Core Collection (Clarivate), IEEE Xplore, and ACM Digital Library, covering records from inception to July 5, 2024. Search filters were used to remove studies on children and animals when possible. The results were limited to the English language. Table 2 provides a subset of unique search keywords used for database queries. The complete search strategy and associated keywords for databases are detailed in Multimedia Appendix 2. The search results were exported as RIS files, consolidated, imported into the Covidence web application for literature review, and deduplicated. The reference lists of the included studies were also examined to identify any additional relevant studies.

**Table 2:** A subset of the unique search keywords used to search the databases.

Concept	Sample keywords
Cardiovascular diseases	Cardiovascular Diseases, Heart Diseases, Auriculo-Ventric, Irregular Heart Rate, Small Vessel Disease, Cardiomyopath, Thrombophlebit, High Blood Pressure, Stroke, Heart Attack
Real time	Real-Time, Live, Online, Dynamic, Synchronous, Streaming, Simultaneous, Cloud, Fog, Edge, Continuous
Artificial intelligence	Artificial Intelligence, Pattern Recognition, Decision Trees, Supervised Learning, Natural Language Processing, Large Language Model, ChatGPT, Transformer Architect, Neural Network, Intelligent System, Deep Learning
Wearable devices	Wearable Devices, Remote Sensing Technology, Internet of Things, Wearable, Smart sensor, Garment, Smart eyewear, Smart jewellery, Electronic Textile, Electronic Skin, FitBit

### Selection of Sources of Evidence

A group of 4 independent reviewers (AV, DJ, JK, and ZH) conducted the title and abstract screening using the Covidence web application. Each study was independently reviewed by at least 2 of these reviewers. Relevant studies identified during this process underwent full-text review and data charting, performed by at least 2 reviewers from the same group (AV, DJ, JK, and ZH). Any conflicts arising during the title and abstract screening or full-text review were resolved by AA.

### Data Charting Process and Data Items

To address the research questions for this scoping review, a data-charting form was designed to extract relevant information from the reviewed studies. The form consisted of five sections: (1) study characteristics, participants, and settings, as well as cardiovascular condition under monitoring; (2) study aims, methodologies, and key findings; (3) characteristics of wearable devices; (4) characteristics of real-time AI algorithms; and (5) robustness analysis of AI algorithms.

### Synthesis of Results

To address the research questions, a descriptive analysis was performed, followed by a narrative summary of relevant study characteristics presented using tables. Studies were analyzed based on the data characteristics outlined in the previous subsection. Due to the heterogeneity in populations, CVDs, outcome measurement tools, and measurement times, a meta-analysis was not conducted [29].

## Results

### Selection of Sources of Evidence

**Figure 1:**
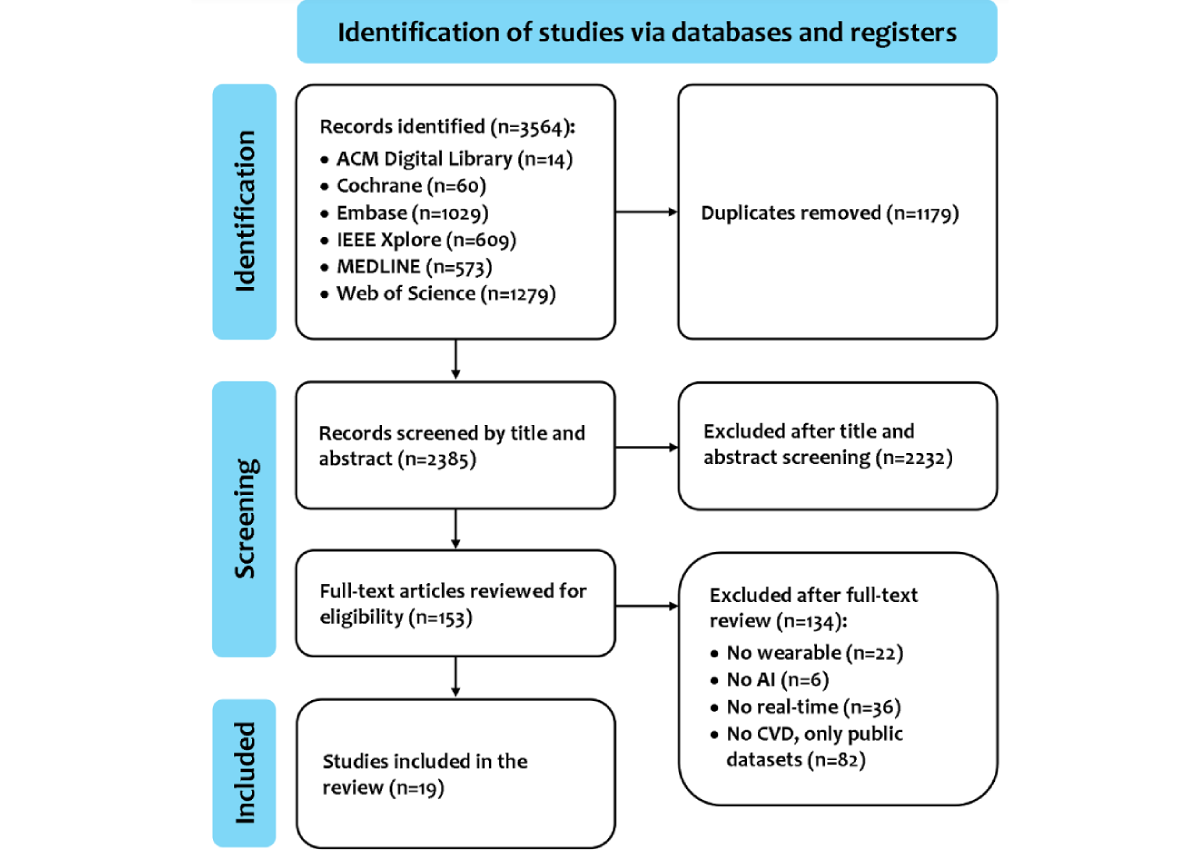
PRISMA (Preferred Reporting Items for Systematic Reviews and Meta-Analyses) ﬂow diagram for the scoping review. Of the 2385 unique titles and abstracts initially screened, 153 full-text studies were further evaluated, resulting in 19 studies being included in the scoping review. ACM: Association for Computing Machinery; AI: artificial intelligence; CVD: cardiovascular disease; IEEE: Institute of Electrical and Electronics Engineers.

Figure 1 illustrates the PRISMA (Preferred Reporting Items for Systematic Reviews and Meta-Analyses) flow diagram, which describes the study selection process. Upon removing duplicates, a total of 2385 studies were identified through comprehensive literature searches of electronic databases. Following title and abstract screening, 2232 studies were excluded, and 153 full-text studies were retrieved for full-text review. Among these 153 studies, 134 were excluded due to the absence of 1 or more of the 4 aforementioned inclusion criteria. This resulted in the inclusion of 19 studies.

### Characteristics of Sources of Evidence

Multimedia Appendices 3-7 show the 5 aforementioned categories of data items for the studies included in this scoping review. A “—“ in the tables indicates that the respective item was not mentioned or discussed in the paper.

### Characteristics of Studies

As outlined in Multimedia Appendix 3, the included studies (n=19) were published between 2010 and 2024, with the majority, 17 (89.5%), published between 2019 and 2024 [30-46], highlighting the increasing shift toward real-time AI solutions for monitoring cardiovascular conditions. Among the included studies, 5 (26.3%) were conducted in the United States [32,40,44,45,47], 4 (21.1%) in Taiwan [33,35,36,48], 3 (15.8%) in China [31,34,46], 2 (10.5%) in India [37,43], and 1 (5.3%) each in Japan [30], Turkey [38], Pakistan [39], Bangladesh [41], and Sri Lanka [42]. Of the included studies, 14 (73.7%) were journal papers, while 5 (26.3%) were peer-reviewed conference publications.

### Characteristics of Participants

One of the inclusion criteria for this scoping review was that the selected studies must have involved the collection of data from adult participants specifically for monitoring cardiovascular conditions. Consequently, a significant number of studies were excluded during the title and abstract screening, as well as the full-text review (n=82). The majority of these excluded studies focused on the development and evaluation of AI algorithms or AI-driven platforms for cardiovascular condition monitoring using publicly available datasets, such as the Massachusetts Institute of Technology-Beth Israel Hospital (MIT-BIH) Arrhythmia Database [49]. Such studies, while valuable for algorithmic advancements, do not provide insights into the feasibility and practical challenges associated with deploying AI models for real-time cardiovascular condition monitoring in real-world settings.

The included studies generally lacked comprehensive details regarding the demographic information of participants whose data were collected and analyzed for cardiovascular condition monitoring. Notably, as can be seen in Multimedia Appendix 3, 7 out of the 19 included studies did not provide any participant demographic information [31,33,34,36,39,43,47]. A highly variable number of participants was reported in the 12 studies that disclosed participant counts, ranging from as few as 2 participants [38] to as many as 350 participants [46]. For studies that reported sex distribution, the balance varied. Some studies included more females [30,32,46], while others reported more males [40,44,45]. Age ranges also varied significantly across studies, with the youngest participants reporting having a mean age of 33.2 years [30] and the oldest having a mean age of 72.1 years [45].

Depending on the study design, some studies included only those participants with CVD [31,33-37,40,41,43-47], others involved a mixed population of both healthy individuals and those with CVD [32,38,42,48], and 1 study included only healthy participants [30]. With the exception of Colombage et al [42], who monitored participants with both heart failure and diabetes, all other studies focused exclusively on monitoring 1 or more cardiovascular conditions without accounting for comorbidities beyond CVDs.

### Characteristics of Study Settings

Unexpectedly, with the exception of a few studies [32,43-45], which reported study durations of 1, 2, 4, and 16 weeks, the other studies involved data collection lasting only for 1 day [33,46] or a single session [35,40,48]. In addition, while 3 studies were conducted in home settings [30,32,44], all other studies took place in hospital or laboratory environments.

As outlined in Multimedia Appendix 3, in 84.2% (16/19) of the studies, the cardiovascular conditions under monitoring predominantly involved the detection of various types of cardiac arrhythmias, with a primary focus on atrial fibrillation (AF) [30-36,38,41,43-48]. Among these, 2 studies additionally measured the AF burden [32,44], and 1 study aimed to predict AF by detecting early warnings or the onset of AF [46]. One study concentrated on the measurement of left ventricular ejection fraction [40]. In addition, 2 studies focused on detecting heart disease [37] and heart failure [42], while 1 study investigated the prediction of cardiac arrest [39].

### Characteristics of Wearable Devices

Depending on the data modalities required for AI-driven cardiovascular condition monitoring, primarily ECG data, a variety of wearable devices were used in the included studies. As outlined in Multimedia Appendix 5, the studies either developed custom hardware sensors or used commercially available wearable devices, such as Polar [30], Samsung [32], Xiaomi [42], Verily [44], and Apple [45] smartwatches. Typically, studies that collected data over extended periods (at least 1 week) relied on commercial smartwatches suitable for everyday use, whereas those conducted in controlled laboratory settings used custom-built hardware wearable sensors [33-40,48].

Seven out of 19 (36.8%) studies used only ECG signals [31,33,34,36,43,46,48], while another 7 (36.8%) studies incorporated ECG signals along with additional physiological or motion signals, such as PPG, acceleration, blood pressure, galvanic skin response, skin temperature, and oxygen saturation [32,39-41,44,45,47]. Other studies exclusively used non-ECG signals, such as PPG [35], heart rate, skin temperature, and acceleration [37], or electrical signals from skin-muscle interfaces [38]. The data collection frequency ranged from every hour [42] to 1200 Hz [33]. Either raw signals or features extracted from signals were input to AI models for analysis.

Of the 19 included studies, 8 (42.1%) deployed AI models directly on wearable devices for data analysis and inference [31,32,35,37,40,44,45,48]. Other studies transferred wearable sensor data to a cloud for AI-based analysis [33,34,39,41-43], while a few used a hybrid approach, deploying AI models on both wearable devices and the cloud [35,44].

### Characteristics of AI Algorithms

As outlined in Multimedia Appendix 6, except for 2 studies that performed prediction tasks, one predicting the early warning or onset of AF [46] and another predicting the risk of cardiac arrest [39], all other studies focused on detection. Except for 3 studies using supervised AI models for regression tasks to measure left ventricular ejection fraction [40] and AF burden [32,44], other studies used supervised AI models for classification. These classification tasks varied, including binary classifications (eg, normal vs abnormal cardiovascular conditions) [31,32,35,37,38,43,45] and categorical classifications distinguishing between multiple cardiovascular conditions [30,33,34,36,41,42,44,46-48].

Most of the included studies used preexisting machine learning and deep learning models rather than developing novel AI architectures. The machine learning models applied included rule-based classifiers, logistic regression, decision tree, k-nearest neighbor, random forest, gradient-boosting machines, support vector machine (SVM), feed-forward neural networks, and hidden Markov model [30,32,36,37,39,42,47,48]. The deep learning models primarily comprised low-complexity 1D or 2D Convolutional Neural Networks (CNNs) [31,33-35,38,40,43,45,46], with a few studies incorporating architectures such as residual layers [44] and attention mechanisms [41]. In machine learning models, feature extraction is a separate step where meaningful characteristics are manually engineered from raw wearable data before being input into the model for analysis. In contrast, deep learning models, such as CNNs, take raw data directly as input. These models automatically learn and extract relevant features through their convolutional layers during training, eliminating the need for manual feature engineering. However, deep learning models are typically more complex, with higher computational demands and longer inference times, which is a key consideration for real-time cardiovascular monitoring.

The annotation of wearable sensor data for supervised AI model development was predominantly performed by cardiologists in most studies. However, some studies used public datasets, such as the MIT-BIH Arrhythmia Database [49], for training AI models, which were subsequently tested on data collected from their study participants [30,31,33,37,41,45,47]. Depending on the specific task, a variety of evaluation metrics were used to assess the performance of AI algorithms, with accuracy being the most commonly used.

### Synthesis of Results

#### Overview

Research question 1: What are the primary challenges of AI-driven platforms for real-time cardiovascular condition monitoring with wearable devices, and how are these challenges addressed?

Several challenges associated with AI-driven platforms for real-time cardiovascular condition monitoring using wearable devices were identified. This section discusses the identified challenges along with strategies proposed in the reviewed studies that addressed or explored potential solutions to mitigate their impact and improve the performance of cardiovascular condition monitoring.

#### Latency in Decision-Making

A significant limitation of deploying AI-driven platforms for real-time cardiovascular monitoring is the delay in making inferences about cardiovascular conditions. This delay arises from computational latency, or *inference time*, which refers to the time required for machine learning and deep learning models to extract features and analyze data using pretrained algorithms. In addition, some platforms implement near real-time inference, where data analysis occurs intermittently rather than continuously. Near real-time analysis is often used to balance clinical relevance with computational efficiency, reducing battery consumption and minimizing web-based data usage when data transfer is required for cloud-based processing. This is especially critical for wearable devices, as battery depletion leads to data gaps and interrupted monitoring. For example, the platform developed by Zhu et al [32] processes data in 5-minute intervals to address these trade-offs. However, they noted that such intervals are insufficient to capture brief arrhythmic episodes that occur within shorter time frames, presenting a significant limitation.

As outlined in Multimedia Appendix 6, AI inference times varied widely in the included studies, ranging from milliseconds to several seconds, depending on the platform and implementation. The fastest reported inference time was 100 milliseconds [46], followed by times under a second [30,31,47], 2 seconds [39], less than 5 seconds [33], and under 6 seconds [48]. Inference frequencies, which often correspond to the size of data window lengths, also differed significantly in the included studies. These include processing every second [30,47], every 5 seconds [45], and every 15 seconds [46]. These variations highlight the trade-offs AI platform developers must consider, balancing real-time responsiveness, computational demands, and energy efficiency on wearable and cloud-based platforms.

#### Motion Artifacts

Motion artifacts are a significant challenge in wearable-based cardiovascular monitoring, particularly for ECG and PPG-based platforms, as they introduce noise that disrupts the detection of true physiological signals. Commonly caused by user movements or improper sensor-skin contact during everyday activities or dynamic conditions such as exercise, these artifacts degrade the signal-to-noise ratio and often overlap with the frequency range of cardiac signals [32]. This overlap complicates the differentiation between true heart rhythms and noise, compromising accurate rhythm detection and increasing the likelihood of missed detections. In addition, preprocessing to filter motion artifacts and noise introduces further latencies, particularly during periods of high physical activity when signal corruption is prevalent.

Hu et al [47] addressed motion artifacts by integrating accelerometer data to classify user activity and differentiate artifacts from true ECG signals. Ye et al [31] optimized motion artifact management by reconstructing multicycle ECG segments to retain correlation features. In the platform developed by Nguyen et al [35], motion artifacts were addressed through a hybrid deep neural network, where a 1D CNN filtered out low-quality PPG signals caused by motion or ambient light interference, ensuring that only reliable data were forwarded to a 2D CNN for the primary task of AF detection.

#### Participant Compliance

Adherence and participant compliance remain critical challenges in wearable-based cardiovascular monitoring. Participants often face difficulties consistently recharging the device on time, wearing it, and maintaining proper sensor-skin contact, which is essential for obtaining reliable PPG and ECG data. While features such as battery recharge reminders and sensor contact monitors can help notify users of low battery or improper device wear, compliance rates have shown significant variability [32]. Notifications must also be limited to prevent overwhelming participants, which can sometimes delay necessary corrections. In addition, adherence variability has been linked to demographic differences and individual behaviors, underscoring the importance of developing personalized notification systems to improve compliance [32].

Poh et al [44] highlighted participant compliance, reporting a median device wear time of 18.3 hours daily. Compliance was supported by automated notifications prompting users to perform ECG acquisitions when AF was detected through continuous PPG. These notifications encouraged consistent use and improved data quality for continuous monitoring in real-world ambulatory environments.

#### Internet and Bluetooth Connection

Maintaining reliable internet and Bluetooth connections is a significant challenge for wearable-based monitoring systems, especially for platforms requiring data transfer to the cloud for AI-based analysis or to a base device, such as a smartphone or computer, acting as a hub [30]. Interruptions or instability in these connections can lead to delays, data loss, or incomplete uploads, compromising the system’s effectiveness and accuracy.

#### Battery Life

Battery life is a critical challenge in wearable-based monitoring, as the need for frequent charging directly impacts usability and participant adherence [32]. While notifications can remind users to recharge devices, particularly to ensure the collection of overnight data, maintaining consistent compliance remains difficult. Participants often forget or neglect to charge or wear the device regularly, resulting in data gaps that undermine the system’s ability to provide effective continuous monitoring.

To enhance battery life, the wearable sensor node developed by Hu et al [47] integrated energy-efficient hardware components and low-power Bluetooth communication modules, enabling 24-hour monitoring with a 500-mAh battery. Reliable data transmission was ensured through Bluetooth Low Energy to a smartphone gateway, where AI-based analyses were conducted. Ye et al [31] addressed battery limitations in their wearable cardiac arrhythmia–monitoring processor using an ultra–low-power design.

Research question 2: How are AI algorithms developed to support robust cardiovascular condition monitoring?

Robustness, in the context of AI for cardiovascular monitoring, refers to an algorithm’s ability to sustain performance under variable, imperfect, or unforeseen conditions. Balendran et al [50] conducted a scoping review to explore robustness concepts in the application of machine learning models in health care. They identified 8 key robustness concepts, which are detailed in the following subsections and outlined in Multimedia Appendix 7, highlighting whether they were addressed in the reviewed studies. While all 8 concepts are considered in this section, the eighth concept is not included in Multimedia Appendix 7, as none of the included studies addressed it.

#### Input Perturbations and Alterations

Perturbations such as noise in data collected from wearable devices (eg, ECG and PPG) are often caused by factors such as motion artifacts from body movements. These alterations were addressed in the methodologies of the reviewed studies, including applying signal-processing techniques such as filtering, using acceleration data to compensate for motion, or analyzing ECG and PPG signals only when motion artifacts were absent. However, the extent to which the reviewed studies addressed these artifacts was limited to the methodology level, without comprehensively validating their platforms against the full range of alterations that can occur continuously in everyday life.

#### Missing Data

Missing input data, such as robustness to random and nonrandom data missingness, is a critical challenge in AI-driven platforms relying on wearable devices. Missing data frequently occurs due to various reasons, including participants not wearing the devices, devices being out of charge, or lack of internet or Bluetooth connectivity [51,52]. However, the reviewed studies did not address this robustness concept on their proposed platforms. To minimize missing data, participant adherence must be improved by encouraging consistent wearing, regular charging, and maintaining internet or Bluetooth connectivity for wearable devices. In addition, when missing data occur, imputation strategies [53] should be used to mitigate their negative impact on the performance of AI algorithms [54].

#### Label Noise

Label noise refers to the uncertainty or inaccuracies in the labels used to train AI models, such as inconsistencies arising from differing expert opinions [55]. Some of the reviewed studies used publicly available datasets to train their AI models and then applied these trained models for cardiovascular condition monitoring on their recruited participants. However, these studies failed to examine label noise caused by discrepancies between the public datasets and the participant-collected data. For example, Pramukantoro and Gofuku [30] trained their models on the MIT-BIH dataset [49] collected in a hospital setting and deployed them for monitoring participants in home environments. In addition, while some studies reported annotating their datasets with cardiologists, they did not provide detailed information about the annotation protocols [56], such as interrater agreement among annotators, or analyze how annotation variability impacted dataset quality and AI model performance.

#### Imbalanced Data

Imbalanced data refers to scenarios where samples from 1 or more classes are significantly underrepresented compared with others [57]. This issue is critical when the minority class represents important events, such as the occurrence of cardiac arrest versus nonoccurrence, or in regression problems, where underrepresented values, such as very low heart rates, are of interest. If left unaddressed, AI models trained on such data are prone to overfitting to the majority class, resulting in the failure to detect or predict crucial cardiac events [58]. While some studies acknowledged and addressed this issue using techniques such as the Synthetic Minority Oversampling Technique or weighted loss functions favoring minority classes [30-32,42,47], the majority of the included studies failed to discuss or mitigate this challenge, limiting the robustness of their models. Recent advancements such as contrastive learning [59] and in-context learning [60] offer promising avenues for improving model performance under imbalanced conditions by leveraging richer representations and context-aware adaptation.

In the context of imbalanced datasets, reliance on accuracy alone can be misleading, as it may reflect performance on the majority class while obscuring poor classification of the minority class. Metrics more appropriate for imbalanced scenarios include the area under the precision-recall curve, area under the receiver operating characteristic curve, *F*_1_-score, precision, recall, specificity, true negative rate, and false-negative rate. These metrics provide a more comprehensive assessment of model performance, particularly in high-stakes applications such as cardiovascular monitoring, where failure to identify rare but critical conditions can lead to serious consequences.

#### Feature Selection

Using feature selection techniques on features extracted from wearable data allows models to focus on the most significant and relevant features for inference in cardiovascular conditions [61]. Selecting the most important features and removing redundant ones enhance model generalizability and reduce overfitting. In addition, reducing the number of features helps avoid the curse of dimensionality, making AI pipelines lighter and more efficient for real-time inference. Among the reviewed studies, none explicitly implemented feature selection techniques; instead, they used either raw sensor data or extracted features directly as input to AI models. One study used attention mechanisms in CNNs [41], enabling the network to focus on specific portions of input data or features that were more effective at distinguishing between classes.

#### Model Specification and Learning

It pertains to robustness against variability arising from the selection, parameterization, and training of AI models [62]. A few of the included studies addressed this robustness concept by using various techniques. For instance, some studies used multiple machine learning algorithms or different versions of the same algorithm to enhance platform robustness against model type and parameter variability. Others implemented cross-validation techniques during the development and validation phases of their AI algorithms to ensure stability and reliability in their performance.

#### External Data and Domain Shift

This robustness concept involves evaluating a model’s performance across diverse populations, tasks, and care settings, such as different environments, age groups, sexes, and ethnicities [63,64]. It is linked to bias, as models trained on homogeneous datasets may not generalize well to underrepresented groups [64]. Ensuring robustness helps mitigate bias and improve fairness and reliability in real-world applications. The distribution and diversity of demographics varied across the reviewed studies. Among those that reported demographic information, the data are often imbalanced, introducing bias into AI models that skew inferences toward majority age groups, sexes, ethnicities, or health conditions. It is crucial to include a representative proportion of demographics during the training process, ensuring alignment with the populations where the AI models will be deployed in real-world settings.

#### Adversarial Attacks

Adversarial attacks involve deliberate alterations to input data to manipulate the predictions of a model [65]. In wearable cardiovascular monitoring, such attacks could subtly distort ECG or PPG signals, leading to misclassification of arrhythmias or missed detection of conditions such as AF. These small changes, invisible to humans, can severely impact model performance. However, none of the reviewed studies addressed or evaluated the potential impact of adversarial attacks on their AI models.

Research question 3: How are AI algorithms and deployment pipelines optimized to enable real-time cardiovascular condition monitoring?

Methods for optimizing AI algorithms and deployment pipelines to enable real-time cardiovascular condition monitoring are examined in this section. Strategies proposed in the reviewed studies, particularly those that explored optimization techniques, are discussed in relation to their effectiveness in improving platform performance, computational efficiency, and real-world applicability.

#### AI Algorithm Optimization

Zhu et al [32] optimized their AI algorithm for real-time inference through a hybrid decision model that combined machine learning classifiers for clean data with statistical heuristics for noisy data. Advanced signal-preprocessing techniques, including noise filtering and motion artifact detection, enhanced robustness during real-world use. The model processed 5-minute data intervals to balance computational efficiency with accuracy, achieving high AF detection accuracy. Pramukantoro and Gofuku [30] demonstrated the use of lightweight machine learning models, enabling inference times under 1 second for real-time cardiovascular monitoring. Ye et al [31] implemented an event-driven neural network architecture, where data analysis is triggered only when predefined signal thresholds are reached. This selective activation minimizes unnecessary computation, enabling efficient real-time processing. Mary et al [43] used modular linear discriminant analysis for dimensionality reduction in their ECG classification platform. Modular linear discriminant analysis extracted the most relevant shape, texture, and statistical features from ECG signals, reducing dimensionality while retaining critical information. This process enhanced their deep neural network’s efficiency and accuracy in real-time analysis.

#### Backend or Server-Side Processing

Hu et al [47] optimized AI deployment by running arrhythmia classification algorithms on a smartphone gateway rather than a wearable device. The smartphone processed ECG data transmitted via Bluetooth, enabling real-time inference through a layered hidden Markov model, reducing computational demands on the wearable and enhancing overall system efficiency and scalability. Lin et al [33] used a wearable ECG device with Bluetooth Low Energy to transmit real-time data to a smartphone app, which provided immediate classification of ECG signals as normal or abnormal. For detailed analysis, the data were transmitted to a cloud server via Wi-Fi or mobile internet, enabling remote, high-precision classification and storage in a cloud database. This 2-tiered system ensured seamless integration of on-device and cloud-based inference. Nguyen et al [35] used a 1D CNN deployed on the wearable device for quality assessment, which evaluated PPG signals in real time to determine whether they met the required standards for further analysis, while a 2D CNN in the cloud performed the main task of AF detection.

#### Hardware Efficiency Improvements

Zhu et al [32] designed their system to focus on efficient data handling and real-time inference while minimizing computational and energy demands, enabling seamless monitoring in diverse settings. Ye et al [31] reported that their system achieved a rapid 0.2-second response time and significantly reduced energy consumption to 842 nW in classification mode.

## Discussion

### Principal Findings

In this scoping review, 19 studies were identified that reported the development or utilization of AI-driven platforms incorporating wearable devices for real-time cardiovascular condition monitoring. These studies leveraged various wearable sensors to acquire multimodal physiological data, which were subsequently processed by AI algorithms to detect or predict cardiovascular events and conditions. Previous reviews have separately demonstrated the feasibility and success of AI-based [18,20-22,25] wearable [10,12-17,20-22] platforms for cardiovascular monitoring, as well as real-time monitoring [23-25] in general health care applications. Building upon these findings, the current review comprehensively integrates AI, wearable technology, real-time capabilities, and cardiovascular conditions to investigate the state of research in this area. The findings align with earlier literature in supporting the promise of such integrated platforms, while also revealing critical areas that remain underexplored, particularly regarding generalizability, robustness, and real-world deployment.

The included studies were conducted across various countries and study settings, ranging from controlled laboratory environments to real-world clinical and home-based contexts. Participant information varied in completeness, with several studies lacking consistent reporting on age and sex distributions. Most studies focused on detecting AF, suggesting a limited exploration of other cardiovascular conditions. A range of wearable devices was used, including commercial systems and custom-built prototypes, capturing physiological data from modalities such as ECG, PPG, and motion sensors. The AI models used spanned from traditional machine learning techniques, such as SVMs and decision trees, to more complex architectures, such as CNNs equipped with attention mechanisms and hybrid models. While most studies reported favorable performance metrics, such as high accuracy, sensitivity, or specificity, these metrics were not always uniformly provided. Moreover, considerations of robustness were often underrepresented, with only a few studies addressing input noise, missing data, class imbalance, or domain shift through approaches such as data augmentation, weighted loss functions, or external validation. These observations underscore the variability in methodological quality across studies and highlight areas where important limitations and challenges remain to be addressed.

### Limitations and Future Recommendations

#### Platform Validation in Real-World Settings

A wearable sensor platform designed to monitor cardiovascular conditions, particularly those critical enough to risk rehospitalization or death if unmanaged, requires thorough performance evaluation. Furthermore, its validation in real-world settings is essential before being implemented for clinical use with participants. This is especially important when the platform is intended for continuous use in community settings, where it must reliably operate over extended periods. The need for validation becomes even more critical when the platform aims to provide real-time inferences about cardiovascular conditions, as this demands high accuracy, low latency, participant compliance, and reliability under dynamic, real-world conditions. There is a significant gap in the current research literature regarding the validation of proposed platforms in real-world settings, highlighting the need for further studies to assess and improve their performance, reliability, and scalability under practical conditions.

#### Lack of Evaluation on Real-World Participants

Many studies were excluded during screening because they relied solely on publicly available datasets, such as subsets of the MIT-BIH dataset [49], without recruiting participants for primary data collection. As a result, these evaluations do not assess the platform’s usability, comfort, or effectiveness on actual users. For instance, a deep learning model may demonstrate high accuracy for AF detection using benchmark datasets but may not account for variability in signal quality, user behavior, or device adherence observed in real-world participants. Similarly, while custom ECG hardware may perform well in technical terms, it might be unsuitable for continuous wear due to discomfort or practical limitations when tested on real individuals.

#### Lack of Evaluation in Real-World Environments

Most included studies conducted testing in controlled hospital or laboratory settings, with only a few evaluating their platforms in unsupervised community environments [30,32,44]. However, understanding the effectiveness and usability of AI-driven wearable platforms requires deployment in real-world settings, where participants live independently and are not overseen by researchers or clinicians. Real-world environments introduce critical factors such as motion artifacts, inconsistent device usage, battery constraints, and variable connectivity, all of which affect platform reliability. Importantly, cardiovascular events often occur outside clinical settings [66], reinforcing the need for evaluations that reflect these real-use conditions.

#### Lack of Platform Usability, Acceptability, and Adherence Validation and Co-Designing

The reviewed studies did not sufficiently evaluate their platforms in terms of usability, acceptability, and adherence from the perspectives of key stakeholders, including patients, caregivers, and clinicians [67,68]. Important factors such as the physical characteristics of the wearable devices (eg, shape, size, and weight), patient adherence to wearing the devices consistently, the frequency and type of alerts (such as cardiac arrest warnings), and the usability of companion smartphone or web apps require co-design with end users to ensure successful deployment and acceptance in real-world settings [69,70]. A major consideration in improving real-world usability is the frequency and reliability of platform-generated alerts, which are closely tied to the robustness of the underlying AI algorithms. High false-positive rates can lead to alert fatigue [71], causing users to lose trust and potentially ignore even critical alarms.

To mitigate these risks, future work should incorporate participatory design approaches and iterative feedback from patients, caregivers, and clinicians [69]. Continuous involvement of stakeholders in the development and postdeployment refinement of platforms can improve usability, foster trust, and increase adherence, thereby supporting successful real-world adoption.

### Limitations in the Development and Validation of Real-Time and Robust AI Algorithms

#### Trade-Offs Between Shallow and Deep Learning Models for Real-Time Cardiovascular Monitoring

Most of the reviewed studies used traditional machine learning models, such as decision trees, SVMs, and lightweight CNNs. These models were favored due to their lower computational demands and ability to support real-time inference, which is essential for timely cardiovascular condition monitoring. In many cases, shallow models demonstrated comparable performance metrics to deep learning architectures, raising the question of whether increased model complexity is justified. From a deployment perspective, especially on edge devices, simpler models offer advantages in terms of lower latency, reduced power consumption, and ease of integration. Nevertheless, recent advancements in AI research have introduced more sophisticated architectures, such as Transformers [72], which have demonstrated improved performance in analyzing sequential data, such as ECG and PPG signals. These models are capable of capturing long-range temporal dependencies, which can be valuable in detecting subtle patterns in cardiovascular signals. However, their high computational cost often limits their use in real-time or on-device scenarios unless optimization techniques are applied. Approaches such as model pruning, quantization, and knowledge distillation can be used to reduce model size and inference time [73], making these architectures more suitable for deployment in resource-constrained environments.

#### Edge Versus Cloud Deployment Models for AI-Enabled Cardiovascular Platforms

While edge deployment supports on-device processing with minimal delay, several studies used offloading techniques, sending data to smartphone gateways or cloud servers for analysis [74]. Despite the potential of cloud infrastructure for scalable, low-latency AI deployment, modern cloud-based tools specifically designed for AI, such as Google Cloud Vertex AI, AWS SageMaker, and Azure AI [75], were not used in the reviewed studies. These platforms provide capabilities for efficient training, deployment, and maintenance of AI models, including support for real-time inference and compliance with health care data standards. Their use could significantly enhance the flexibility, performance, and maintainability of AI-enabled cardiovascular monitoring systems. Future studies should consider the trade-offs between edge and cloud deployment models when designing AI-driven platforms. While edge computing ensures privacy and immediate feedback, cloud deployment enables more complex models and centralizes updates and maintenance. Both deployment strategies involve specific considerations around latency, energy consumption, data privacy, and reliability, which must be evaluated based on the target application and environment.

#### Robustness and Ethical Considerations in AI Algorithms for Cardiovascular Monitoring

The robustness of the included studies was examined under research question 3, based on the 8 robustness dimensions defined by Balendran et al [50]. Input perturbations, such as motion artifacts or physiological noise in ECG and PPG signals, were minimally addressed. Some studies applied basic signal filtering, but few used advanced noise mitigation strategies. Missing data, often resulting from sensor detachment or transmission issues, were rarely discussed. Models that ignore such gaps risk biased outputs. Incorporating imputation methods or continuity-aware algorithms could improve resilience. Data imbalance was common, with normal cardiovascular states vastly outnumbering abnormal ones. Many studies reported only accuracy, which is insufficient for imbalanced datasets. Metrics such as precision, recall, specificity, *F*_1_-score, and area under the precision-recall curve provide a more comprehensive view. External validation was often absent, raising concerns about generalizability and algorithmic bias when models are applied across diverse populations or devices.

Adversarial robustness, although critical, was not explicitly explored. Wearable data can be susceptible to manipulation or distortion. To mitigate such vulnerabilities, techniques such as adversarial training or input preprocessing can be used [76]. Cloud-deployed models were typically supported by encrypted data transfer and Health Insurance Portability and Accountability Act–compliant storage. However, more advanced privacy-preserving strategies, such as federated learning and split learning with differential privacy, offer better protection by avoiding transmission of raw data [77]. These approaches enable collaborative model training across devices, sharing only model parameters or components.

Interpretability was another underaddressed dimension. Machine learning and deep learning models, including CNNs and Transformers, are often treated as black boxes. To promote clinical adoption, interpretability techniques should be applied. Tools such as attention mechanisms [72], Grad-CAM [78], and SHAP [79] can help identify critical signal segments or features contributing to a prediction. This allows clinicians not only to detect cardiovascular conditions but also to understand potential underlying causes, supporting more informed decision-making.

Together, these observations highlight the need for more robust, transparent, and ethically aware AI systems. Addressing these robustness dimensions is essential for advancing AI-driven cardiovascular monitoring platforms that are safe, reliable, and applicable in real-world health care settings.

### Other Methodological and Reporting Limitations

Among all cardiovascular conditions requiring real-time monitoring, the included studies addressed only a limited subset, with most focusing on AF detection. Critical conditions, such as stroke, remain unexamined. Many studies also lacked essential methodological details, including complete demographic information [31,33-39,41,43,47], study duration [30,31,34,36-39,41,42,47], and data collection frequency [36,38,39]. These omissions limit the interpretability and reproducibility of findings.

There is also inconsistency in how AI algorithms are applied to wearable data for cardiovascular monitoring. Although continuous data collection and inference are desirable, practical constraints such as battery life, network reliability, and participant adherence often necessitate compromises. To determine appropriate monitoring frequency and system design, collaboration between platform engineers and cardiovascular clinicians is essential, as requirements vary across conditions, such as AF detection, cardiac arrest detection, and early warning applications.

### Methodological Limitations and Scope of the Review

This review used a systematic and reproducible approach, guided by a well-established scoping review framework. The search strategy was developed in collaboration with a Library Sciences Expert to ensure comprehensive coverage of relevant literature from the inception of the technology to the present. However, some methodological limitations should be acknowledged. The exclusion of non–English language papers may have led to the omission of relevant studies published in other languages. Gray literature and preprints were also excluded, potentially contributing to selection bias. Although multiple databases were searched, some degree of publication bias or incomplete database coverage cannot be ruled out. In addition, the generalizability of findings may be limited due to the predominant focus on AF in the included studies and the underrepresentation of diverse cardiovascular conditions and populations.

### Conclusions

AI-powered platforms integrated with wearable devices show strong potential for transforming real-time cardiovascular condition monitoring in community settings. By leveraging AI algorithms to analyze data from wearable sensors, these systems enable early detection of conditions such as AF and cardiac arrest, allowing timely intervention by clinicians or caregivers. However, most studies reviewed were limited to short-term evaluations in hospital environments, with minimal validation in unsupervised, real-world contexts. Key challenges include inconsistencies in data collection and inference frequency, lack of model robustness evaluation, and limited coverage of ethical considerations such as privacy, interpretability, and algorithmic bias. Few studies addressed robustness dimensions such as resilience to input noise, dataset imbalance, or domain shift, which are critical for safe deployment. Usability and adherence validation across diverse populations remain underexplored, as do issues related to participant compliance, battery constraints, and user-centered design. To advance this field, future research should (1) validate platforms in diverse, community-based settings with long-term use; (2) optimize and test robust AI models under real-world constraints; (3) address usability, acceptability, and co-design with stakeholders; (4) evaluate privacy-preserving techniques and interpretability tools to foster trust; and (5) develop standardized protocols for data collection and model evaluation.

Interdisciplinary collaboration will be essential to enhance the practicality, equity, and reliability of AI-enabled cardiovascular monitoring systems for real-world use.

## Appendix

Multimedia Appendix 1 PRISMA-ScR checklist and explanation.

Multimedia Appendix 2 Search strategy and associated keywords.

Multimedia Appendix 3 Baseline characteristics of the reviewed studies, including study characteristics, participants, and settings, as well as the cardiovascular condition under monitoring.

Multimedia Appendix 4 Aims, methodologies, and key findings of the included studies.

Multimedia Appendix 5 Characteristics of wearable devices in the included studies.

Multimedia Appendix 6 Characteristics of artificial intelligence algorithms in the included studies.

Multimedia Appendix 7 Robustness analysis of the artificial intelligence algorithms in the included studies.

## Abbreviations

AF: atrial fibrillation
AI: artificial intelligence
CNN: Convolutional Neural Network
CVD: cardiovascular disease
ECG: electrocardiography
MIT-BIH: Massachusetts Institute of Technology-Beth Israel Hospital
PPG: photoplethysmography
PRISMA: Preferred Reporting Items for Systematic Reviews and Meta-Analyses
PRISMA-ScR: Preferred Reporting Items for Systematic Reviews and Meta-Analyses extension for Scoping Reviews
SVM: support vector machine
