# Supplementary material for: AI-Driven Real-Time Monitoring of Cardiovascular Conditions With Wearable Devices: Scoping Review
Source: JMIR Mhealth Uhealth. 2025 Nov 11;13:e73846. doi: 10.2196/73846 (PMC12777649; doi:10.2196/73846)
Supplement: Multimedia Appendix 2 [file mhealth_v13i1e73846_app2.docx]

Multimedia Appendix 2. Search strategy and associated keywords.

| # | Query | Results from 5 Jun 2024 |
| --- | --- | --- |
| 1 | exp Wearable Electronic Devices/ | 20,368 |
| 2 | Remote Sensing Technology/ | 4,291 |
| 3 | "Internet of Things"/ | 1,171 |
| 4 | wearable*.tw,kf. | 31,631 |
| 5 | ((activity or fitness) adj3 (tracker* or monitor?)).tw,kf. | 8,418 |
| 6 | hearing aid?.tw,kf. | 11,811 |
| 7 | internet of things.tw,kf. | 7,908 |
| 8 | ((smart or body-borne or bodyborne or body-worn or on body or on the body or wrist-worn or on wrist? or on the wrist? or head-worn or on the head?) adj3 (device* or tech* or sensor* or sensing or gear? or gadget* or electronic*)).tw,kf. | 9,362 |
| 9 | (smartdevice or smarttech* or smartsensor* or smartsensing or smartgear? or smartgadget* or smartelectronic*).tw,kf. | 16 |
| 10 | ((smart or tech* or digital or connected or electronic) adj3 (watch* or glass* or sunglass* or goggle* or eyeware* or eye wear* or ring* or implant* or wristband* or band* or armband* or strap* or shirt* or pant* or cloth* or garment* or underwear* or jacket* or sleeve* or cuff* or short* or sock* or shoe* or mask* or glove* or jeweller* or jewelr* or pendant* or bracelet* or necklace* or pin? or earring* or accessor* or headgear* or headcap* or headset* or headband* or helmet* or belt* or textile* or fabric* or polymer* or lens* or patch* or tattoo* or earbud* or earphone* or headphone*)).tw,kf. | 82,122 |
| 11 | (smartwatch* or smartglass* or smartsunglass* or smartgoggle* or smarteyeware* or smarteyewear* or smartring* or smartimplant* or smartwristband* or smartband* or smartarmband* or smartstrap* or smartshirt* or smartpant* or smartcloth* or smartgarment* or smartunderwear* or smartjacket* or smartsleeve* or smartcuff* or smartshort* or smartsock* or smartshoe* or smartmask* or smartglove* or smartjeweller* or smartjewelr* or smartpendant* or smartbracelet* or smartnecklace* or smartpin? or smartearring* or smartaccessor* or smartheadgear* or smartheadcap* or smartheadset* or smartheadband* or smarthelmet* or smartbelt* or smarttextile* or smartfabric* or smartpolymer* or smartlens* or smartpatch* or smarttattoo* or smartearbud* or smartearphone* or smartheadphone*).tw,kf. | 1,409 |
| 12 | (electronic textile* or e-textile* or digital textile* or intelligent textile* or smartwear or e-uniform*).tw,kf. | 543 |
| 13 | (human-integrated electronic* or epidermal electronic* or electronic skin).tw,kf. | 1,003 |
| 14 | ((head-mounted or head? up or head-worn) adj3 display*).tw,kf. | 2,130 |
| 15 | ((video or VR or augmented reality or AR) adj2 (eyeware or eyewear* or headset*)).tw,kf. | 452 |
| 16 | (accelerometer* or pedometer* or GPS or actigraph*).tw,kf. | 63,779 |
| 17 | ((wrist or watch or belt) adj3 (computer* or phone* or sensor* or monitor*)).tw,kf. | 1,490 |
| 18 | self-monitoring device*.tw,kf. | 106 |
| 19 | ((chest or body-borne or bodyborne or body-worn or on-body) adj3 (sensor* or heart-rate monitor*)).tw,kf. | 615 |
| 20 | ((Apple or Google or Samsung or Sony or Garmin or Withings or Polar or Huawei or LG or Xiaomi or Pebble) adj3 watch*).tw,kf. | 482 |
| 21 | (ZenWatch* or Zen Watch* or Q Founder or Q Marshal or Q Wander or Q Explorist or Q Venture or Q Control or Q Explorist HR or Q Venture HR or Q Sport or Q The Carlyle HR or Q Julianna HR or Garrett HR or Fossil HR or Pixel Watch* or G Watch* or Watch Urbane* or Tambour Horizon or Michael Kors Access or Michael Kors Runway or Misfit Vapor or TicWatch* or Montblanc Summit or Moto 360 or Moto360 or RunIQ or Nixon Mission or Oppo Watch* or Polar M600 or Galaxy Watch* or GalaxyWatch* or Samsung Gear* or Gear Live or Galaxy Gear* or Skagen Falster or Suunto 7 or Tag Heuer Connected or Wear24 or MiWatch* or Mi Watch* or ZTE Quartz or AppleWatch* or Pebble Time or ScanWatch* or Garmin or Withings).tw,kf. | 505 |
| 22 | (FitBit* or Fit Bit* or Jawbone UP or FuelBand* or Samsung Gear or Empatica or WHOOP or HeartGuide).tw,kf. | 1,768 |
| 23 | (Wear OS or Android Wear or AsteroidOS or watchOS).tw,kf. | 8 |
| 24 | (Google Glass* or Beyond Glasses or EyeTap or SixthSense or Magic leap or Vuzix or HoloLens or Epiphany Eyewear or Moverio or Everysight Raptor or Echo Frame* or Ray-Ban Stories or RayBan Stories or Golden-i or Looxcie or castAR or Airscouter).tw,kf. | 663 |
| 25 | ((Xiaomi adj3 glass*) or (Snap adj3 spectacles) or (Brilliant Labs adj3 Frame*) or (DAQRI adj3 glass*)).tw,kf. | 0 |
| 26 | bionic contact lens*.tw,kf. | 0 |
| 27 | (Oura ring* or McLear ring* or RingPay or NFC Payment Ring* or RingConn or Galaxy Ring).tw,kf. | 60 |
| 28 | (Hexoskin or Project Jacquard or (Google adj3 Jacquard) or (Intel adj3 chromat)).tw,kf. | 26 |
| 29 | or/1-28 [Wearables Concept] | 216,275 |
| 30 | Cardiovascular Diseases/ | 184,665 |
| 31 | exp heart diseases/ or exp pregnancy complications, cardiovascular/ or exp vascular diseases/ | 2,653,804 |
| 32 | ((cardiovascular or cardio* or cardiac or heart or coronary or atrial or ventric* or atrioventric* or auriculo-ventric* or pericard* or myocardi* or endomyocard* or endocard* or cardiopulmonar* or cardio-renal or cardiorenal or renocardiac or valve or valvular or aortic or aorta or mitral or arterial or artery or arteries or vein? or venous or vascula* or carotid or cerebrovascular or hemostatic) adj3 (disease* or disorder* or illness* or dysfunction* or failure* or event? or abnormalit* or decompensat* or syndrome* or defect* or hypertroph* or complication* or insufficien* or accident* or stenos* or prolapse* or thrombos* or thrombus or embolism* or thromboembolism* or obstruct* or occlus* or accident* or fistula* or dissect* or ruptur*)).tw,kf. | 1,586,876 |
| 33 | ((atrial or atrium* or auricular or ventric* or heart* or cardiac) adj3 (fibrillation* or flutter*)).tw,kf. | 125,170 |
| 34 | (irregular heart rate* or irregular heartrate* or irregular heart beat or irregular heartbeat* or irregular heart rhythm* or afib or a-fib).tw,kf. | 1,221 |
| 35 | (arrhythm* or dysrhythm* or bradycard* or bradyarrhythm* or tachycard* or tachyarrhythm*).tw,kf. | 201,803 |
| 36 | (brugada or long QT or QT syndrome* or (QT adj3 prolong*) or torsade? or sick sinus or pre-excitation or preexcitation).tw,kf. | 25,460 |
| 37 | (extrasystole* or parasystole* or asystole*).tw,kf. | 8,041 |
| 38 | (premature adj3 (complex or complices or contraction* or beat*)).tw,kf. | 8,188 |
| 39 | ((atrioventricular or av or a-v or bundle branch or fascicular or interatrial or sinoatrial) adj3 (dissociat* or block*)).tw,kf. | 26,382 |
| 40 | (commotio cordis or cardiac concuss*or cor pulmonale).tw,kf. | 259 |
| 41 | ((cardiac or cardiogenic or heart? or circulat*) adj3 (arrest? or death* or shock?)).tw,kf. | 121,770 |
| 42 | (aortiti* or arterit* or vasculit* or vasculopath* or microangiopath* or small vessel disease* or macroangiopath* or angiopath*).tw,kf. | 100,224 |
| 43 | (cardiac output* or tamponade*).tw,kf. | 65,981 |
| 44 | (cardiomegal* or cardiomyopath* or myocardiopath* or myocardit* or cardit* or cardiotoxic* or endocardit* or pericardit* or pleuropericardit*).tw,kf. | 187,694 |
| 45 | (apical ballooning or broken heart syndrome* or angina or angor pectoris or stenocardia).tw,kf. | 61,439 |
| 46 | (aneurysm* or microaneurysm* or macroaneurysm*).tw,kf. | 150,015 |
| 47 | (atherosclero* or atherogenes* or arterioscleros* or left main disease*).tw,kf. | 206,025 |
| 48 | ((artery or arteries or arterial or venous or vascular or cerebrovascular) adj3 spasm*).tw,kf. | 4,466 |
| 49 | (vasospasm* or angiospasm*).tw,kf. | 13,980 |
| 50 | (infarct* or heart attack* or NSTEMI or STEMI or TIA or stroke? or ischemi* or ischaemi* or apoplex*).tw,kf. | 905,987 |
| 51 | (thrombus or thrombos* or thrombophlebit* or thrombotic or thromboembol* or embolism* or embolic or embolus or atheroembol* or phlebit* or periphlebit* or VTE or atherothromb* or phlebothromb*).tw,kf. | 377,777 |
| 52 | (intermittent claudication* or venous stasis or (raynaud adj3 (disease* or phenomenon* or syndrome*))).tw,kf. | 9,115 |
| 53 | (hypertens* or hypotens* or high blood pressure* or elevated blood pressure* or white coat syndrome* or low blood pressure* or syncope*).tw,kf. | 632,663 |
| 54 | or/30-53 [Cardiovascular Diseases Concept] | 3,921,107 |
| 55 | exp Artificial Intelligence/ | 198,665 |
| 56 | Pattern Recognition, Automated/ | 26,639 |
| 57 | exp Decision Trees/ | 12,724 |
| 58 | (artificial adj3 intelligence*).tw,kf. | 52,513 |
| 59 | AI.tw,kf. | 56,986 |
| 60 | ((comput* or machine) adj3 intelligence).tw,kf. | 4,446 |
| 61 | (computer adj3 (reasoning or vision system or heuristic*)).tw,kf. | 349 |
| 62 | (knowledge adj1 (base? or representation or acquisition)).tw,kf. | 23,331 |
| 63 | (machine adj3 learn*).tw,kf. | 117,583 |
| 64 | ((transfer or deep or hierarchical supervised or semi-supervised or semisupervised or unsupervised or reinforcement or comput*) adj3 learn*).tw,kf. | 88,707 |
| 65 | (support adj3 vector*).tw,kf. | 31,849 |
| 66 | (expert system* or intelligent system*).tw,kf. | 4,844 |
| 67 | interference engine*.tw,kf. | 8 |
| 68 | fuzzy logic*.tw,kf. | 2,876 |
| 69 | natural language process*.tw,kf. | 9,800 |
| 70 | large language model*.tw,kf. | 2,064 |
| 71 | machine translat*.tw,kf. | 367 |
| 72 | (generative pre-trained transformer* or generative pretrained transformer* or GPT* or ChatGPT*).tw,kf. | 9,806 |
| 73 | GPT*.tw,kf. | 6,830 |
| 74 | (neural adj3 network*).tw,kf. | 114,648 |
| 75 | (deep adj3 (network* or model*)).tw,kf. | 36,870 |
| 76 | (artificial adj3 neuron*).tw,kf. | 700 |
| 77 | perceptron*.tw,kf. | 5,481 |
| 78 | connectionist model*.tw,kf. | 522 |
| 79 | generative adversarial network*.tw,kf. | 3,854 |
| 80 | (pattern recogni* adj2 (automat* or system* or comput*)).tw,kf. | 879 |
| 81 | transformer architect*.tw,kf. | 382 |
| 82 | (GenAI or GAI).tw,kf. | 772 |
| 83 | or/55-82 [Artificial Intelligence Concept] | 467,160 |
| 84 | Cloud Computing/ | 1,407 |
| 85 | (live or online or dynamic* or synchronous* or stream? or streamed or streaming).tw,kf. | 1,579,827 |
| 86 | ((active or ongoing or on-going or automat* or continuous* or immediate* or concurrent* or "same time" or "in sync" or synchroniz* or simultaneous* or parallel* or remote* or cloud or edge or mist or fog) adj5 (comput* or network* or service* or storage* or process* or analytic* or analys* or analyz* or data or measur* or record* or input* or predict* or detect* or monitor* or track* or analys* or analytic* or diagnos* or process* or screen* or algorithm* or grade? or grading or method* or decision-making* or suggest*)).tw,kf. | 800,672 |
| 87 | ((cloud or fog or mist or edge) adj3 (comput* or network* or service* or storage* or process* or analytic* or analys*)).tw,kf. | 8,589 |
| 88 | "on cloud".tw,kf. | 556 |
| 89 | or/84-88 [Real Time Concept] | 2,301,670 |
| 90 | 29 and 54 and 83 and 89 | 484 |
