# Supplementary material for: AI-Driven Real-Time Monitoring of Cardiovascular Conditions With Wearable Devices: Scoping Review
Source: JMIR Mhealth Uhealth. 2025 Nov 11;13:e73846. doi: 10.2196/73846 (PMC12777649; doi:10.2196/73846)
Supplement: Multimedia Appendix 3 [file mhealth_v13i1e73846_app3.docx]

Multimedia Appendix 3. Baseline characteristics of the reviewed studies, including study characteristics, participants, and settings, as well as the cardiovascular condition under monitoring.

| Study Characteristics | | | | Study Participants | | | Study Setting | | |
| --- | --- | --- | --- | --- | --- | --- | --- | --- | --- |
| Reference | Year | Type | Country | Female: Male (Total) | Age Mean (Age SD) | Disease | Condition Under Monitoring | Study Setting | Study Duration |
| Lin et al [48] | 2010 | Journal | Taiwan | — (Total = 30) | 71.4 (9.15) | AF and healthy | AF and other rhythm disorders | Hospital | 6 minutes |
| Hu et al [47] | 2012 | Journal | USA | — | — | Arrhythmias | Arrhythmias | — | — |
| Lin et al [33] | 2019 | Conf. | Taiwan | — | — | Arrhythmias | Arrhythmias | Hospital | 24 hours |
| Lin et al [36] | 2019 | Conf. | Taiwan | — | — | Arrhythmias | Arrhythmias | Hospital | — |
| Wasserlauf et al [45] | 2019 | Journal | USA | 9:17 (Total = 26) | 72.1 (7.2) | AF | AF | Hospital | 110 days |
| Zhu et al [32] | 2021 | Journal | USA | 35:6 (Total = 41) | 58.6 (9.6) | AF and healthy | AF and AF burden estimation | Home | 28 days |
| Fu et al [34] | 2021 | Journal | China | — | — | Various CVDs | Arrhythmias and rhythm abnormalities | Hospital | — |
| Ergen [38] | 2021 | Journal | Turkey | — (Total = 2) | — | AF and healthy | AF and heart rate monitoring | Lab | — |
| Pramukantoro and Gofuku [30] | 2022 | Journal | Japan | 4:2 (Total = 6) | 33.2 (3.2) | Healthy | Heartbeat classification into normal vs. abnormal | Home | — |
| Nguyen et al [35] | 2022 | Journal | Taiwan | — (Total = 40) | — | Variants of AF | AF and rhythm analysis | Hospital | 5 minutes |
| Jenifer et al [37] | 2022 | Conf. | India | — (Total = 20) | — | General heart disease | Heart disease | Lab | — |
| Colombage et al [42] | 2022 | Conf. | Sri Lanka | — (Total = 230) | — | Heart failure, diabetes, and healthy | Severity levels of heart failure | Hospital | — |
| Ye et al [31] | 2023 | Conf. | China | — | — | Arrhythmias | Arrhythmia | — | — |
| Howard et al [40] | 2023 | Journal | USA | 27:54 (Total = 81) | 53 (16) | Various cardiac pathologies | Left Ventricular Ejection Fraction | Lab | 3 minutes |
| Islam et al [41] | 2023 | Journal | Bangladesh | — (Total = 22) | — | Arrhythmias | Heart rate and arrhythmias | Lab | — |
| Mary et al [43] | 2023 | Journal | India | — | — | Arrhythmias | Arrhythmias | — | 7 days |
| Poh et al [44] | 2023 | Journal | USA | 50:61 (Total = 111) | 65 (11) | AF | AF and AF burden estimation | Home | 14 days |
| Gavidia et al [46] | 2024 | Journal | China | 189:161 (Total = 350) | 66.5 (14.5) | AF | Onset of AF | Hospital | 24 hours |
| Hannan et al [39] | 2024 | Journal | Pakistan | — | — | Cardiac arrest | Cardiac arrest risk | Hospital | — |
