# Supplementary material for: AI-Driven Real-Time Monitoring of Cardiovascular Conditions With Wearable Devices: Scoping Review
Source: JMIR Mhealth Uhealth. 2025 Nov 11;13:e73846. doi: 10.2196/73846 (PMC12777649; doi:10.2196/73846)
Supplement: Multimedia Appendix 4 [file mhealth_v13i1e73846_app4.docx]

Multimedia Appendix 4. Aims, methodologies, and key findings of the included studies.

| Reference | Aim | Methodology | Key Findings |  |
| --- | --- | --- | --- | --- |
| Lin et al [48] | To develop and evaluate a wearable, wireless, real-time tele-cardiology platform for detecting AF and other rhythm abnormalities, featuring built-in emergency alerts. | Participants were monitored using a lightweight, wireless three-lead ECG device with disposable electrodes. ECG signals were processed locally using an expert system, and abnormal data were transmitted to a remote server via wireless communication. | The platform showed strong performance in detecting AF, supporting early identification, remote monitoring, and timely emergency alerts. |  |
| Hu et al [47] | To develop a real-time platform for continuous monitoring and classification of cardiac arrhythmias. | Wearable ECG sensors were used alongside a layered hidden Markov model to classify arrhythmias based on real-time ECG features and motion data. | The platform achieved high accuracy in arrhythmia detection and demonstrated strong reliability in real-time applications. |  |
| Lin et al [33] | To develop and validate an AI-driven IoT wearable platform for real-time ECG monitoring and arrhythmia detection using a cloud-integrated CNN model. | Clinical trials were conducted at Tainan Hospital using a single-lead wearable ECG patch. Preprocessing included noise and baseline drift removal, followed by arrhythmia classification using a CNN algorithm. | The platform demonstrated reliable real-time arrhythmia detection and classification across both clinical trial data and the MIT-BIH database. |  |
| Lin et al [36] | To develop an AI-driven IoT wearable ECG patch for real-time arrhythmia analysis using decision tree algorithms. | Data were collected from participants using a wearable ECG patch. Features were extracted and analyzed using a decision tree model to detect arrhythmias. | The platform demonstrated reliable arrhythmia detection with efficient real-time performance, supporting its potential for clinical and remote health monitoring. |  |
| Wasserlauf et al [45] | To evaluate the performance of a smartwatch combined with a deep learning algorithm for detecting and quantifying AF episodes and duration, compared to an insertable cardiac monitor. | A CNN was trained and validated using data from participants with implanted cardiac monitors. Participants wore both an AF-sensing smartwatch and an insertable cardiac monitor simultaneously. Data collected included heart rate, activity level, and ECG recordings in real-world conditions. | The system demonstrated high sensitivity for AF detection, effectively identifying longer episodes and overall AF burden. Despite a moderate positive predictive value, it showed strong potential for continuous, noninvasive AF monitoring. |  |
| Zhu et al [32] | To develop and validate wearable-based algorithms for detecting AF and estimating AF burden during daily activities. | Multiple participant groups, including those undergoing elective cardioversion, were monitored using wearable devices. ECG patches were used as reference, while a machine learning algorithm analyzed PPG and accelerometer signals to detect AF and estimate its burden. | The algorithm achieved high sensitivity and specificity for AF detection across diverse populations and settings, with reliable estimation of AF burden during daily activities. |  |
| Fu et al [34] | To develop an AI-enhanced mobile platform for cardiovascular health management with real-time ECG monitoring and diagnostic capabilities. | ECG data were collected using a custom-designed portable device and processed with deep learning models combining CNNs and recurrent neural networks. The models were deployed on the Tencent Cloud for real-time diagnosis. Data annotations were reviewed and refined by cardiologists. | The AI platform demonstrated high diagnostic accuracy and enhanced real-time ECG analysis for cardiovascular health management. |  |
| Ergen [38] | To develop a graphene aerogel-based nano-tattoo for robust heart rate and AF detection during extreme upper-extremity movements. | A nano-tattoo sensor platform was fabricated using graphene aerogel and boron nitride materials. Electrical signals were processed in real time using AI-based neural networks to detect heart rate and AF. | The device demonstrated strong performance in minimizing motion-related noise and accurately detecting AF during various physical activities, outperforming traditional PPG sensors. |  |
| Pramukantoro and Gofuku [30] | To develop a platform for continuous, real-time monitoring of heartbeat patterns to distinguish between regular and irregular beats, supporting early detection of heart-related conditions during daily activities. | Healthy individuals were monitored using a wearable device while sitting or moving around in a real-world setting. The system collected heartbeat timing data and classified each beat into different types. Each person was observed for about 20 to 30 minutes to test the system’s ability to work continuously and in real time. | The system showed high accuracy and reliable performance in identifying different types of heartbeat patterns. It was able to make quick and consistent predictions within one second, supporting its use for continuous monitoring and early detection of potential heart problems. | |
| Nguyen et al [35] | To develop a deep learning model using PPG data for real-time AF detection and rhythm quality assessment. | PPG data were collected from multiple participants and analyzed using deep learning models to assess rhythm quality and detect AF in real time. | The model demonstrated strong performance in both AF detection and rhythm quality evaluation, supporting reliable real-time monitoring and analysis. | |
| Jenifer et al [37] | To design an edge-based IoT device for heart disease prediction, reducing latency and ensuring data privacy. | Sensor data (temperature, pulse rate, and motion) were processed on a Raspberry Pi using a decision tree model trained on a public dataset. Predictions were displayed on an integrated OLED screen. | The platform demonstrated reliable performance on data collected from recruited subjects, supporting effective real-time monitoring and analysis. | |
| Colombage et al [42] | To develop a real-time detection platform for heart failure (including severity), diabetes, and diabetes types using smartwatch data and user-provided information. | Data were collected from smartwatches (e.g., heart rate, blood pressure, respiratory rate) and user inputs (e.g., age, family history). Machine learning models, including logistic regression and random forest, were trained on public datasets and deployed on the cloud for real-time prediction. | The models demonstrated strong predictive performance for heart failure detection, severity assessment, and diabetes classification. Validation on local patient datasets confirmed their reliability in real-world settings. | |
| Ye et al [31] | To develop a low-power, real-time wearable processor for cardiac arrhythmia monitoring that balances generalization performance with computational efficiency. | An event-driven architecture was implemented to process ECG signals, including localization of the QRS complex (the part of the ECG that reflects ventricular depolarization), multi-cycle heartbeat reconstruction, and arrhythmia classification using a neural network. All data were processed locally on a wearable device for real-time analysis. | The processor demonstrated high sensitivity and reliable performance in heartbeat localization, abnormal beat detection, and arrhythmia classification. It operated with ultra-low power consumption (842 nW). | |
| Howard et al [40] | To evaluate the accuracy of a wearable biosensor platform (Cardiac Performance System) using machine learning for automated real-time measurement of LVEF. | The Cardiac Performance System measurements were collected simultaneously using acoustic and ECG sensors. Data were processed by a regression-based neural network. The evaluation included assessments of intraoperator and interoperator variability. | The Cardiac Performance System demonstrated strong performance in estimating LVEF. The model showed high discrimination across LVEF thresholds, with minimal variability both between and within operators. | |
| Islam et al [41] | To develop a deep learning-based IoT framework for remote health monitoring and real-time detection of health issues, enabling proactive healthcare. | Physiological data, including ECG, heart rate, oxygen saturation, and body temperature, were collected using IoT sensors and transmitted to a server via the Message Queuing Telemetry Transport protocol. A pre-trained CNN with attention layers was used to classify health issues and generate reports. | The system accurately classified multiple arrhythmias and detected fever, providing real-time monitoring and timely alerts for physiological abnormalities. | |
| Mary et al [43] | To develop a wearable IoT platform for real-time ECG monitoring and classification of normal versus abnormal heartbeats using adaptive deep neural networks. | ECG data were collected from wearable sensors via Wi-Fi. Signals were preprocessed using denoising, heartbeat alignment, and data standardization. Features were extracted using Modular MLDA and classified using an adaptive deep neural network trained on time-domain and automatically extracted features. | The platform effectively detected abnormal ECG signals with high accuracy. The proposed adaptive deep neural network model outperformed other classification methods, showing superior performance in ECG analysis. |  |
| Poh et al [44] | To validate the performance of a wrist-worn device for detecting AF and estimating AF burden using a PPG-based algorithm. | A prospective, multicenter study was conducted in which participants wore both a wrist-worn Study Watch and a reference ECG patch device simultaneously. | The wrist-worn device demonstrated strong performance in AF detection, showing high sensitivity and specificity, and provided accurate AF burden estimation compared to the reference ECG. |  |
| Gavidia et al [46] | To develop a deep learning model for predicting AF onset at least 30 minutes in advance, enabling early intervention and reducing emergency healthcare demands. | A CNN was developed using 24-hour Holter ECG data for continuous monitoring. The model was validated on external datasets from cohorts in Argentina and France. | The deep learning model successfully predicted AF onset approximately 30 minutes in advance and demonstrated strong performance and generalizability across external datasets. |  |
| Hannan et al [39] | To develop an IoT-based wearable platform for real-time cardiac arrest monitoring and prediction, reducing response time and enabling remote supervision through an Android application. | Real-time data were collected from wearable IoT sensors. Signals were preprocessed, features were extracted, and machine learning models were used for classification. Predictions were compared with trained models, and alerts were transmitted to patients and caregivers via an Android application. | The Random Forest algorithm accurately predicted cardiac arrest and enabled timely alerts with minimal delay through a hybrid IoT–cloud architecture. |  |
