# Supplementary material for: AI-Driven Real-Time Monitoring of Cardiovascular Conditions With Wearable Devices: Scoping Review
Source: JMIR Mhealth Uhealth. 2025 Nov 11;13:e73846. doi: 10.2196/73846 (PMC12777649; doi:10.2196/73846)
Supplement: Multimedia Appendix 5 [file mhealth_v13i1e73846_app5.docx]

Multimedia Appendix 5. Characteristics of wearable devices in the included studies.

| Reference | Type | Model | Data modalities | Data collection frequency | Data transfer frequency | AI algorithm deployed on wearable device | AI inference results delivery method |
| --- | --- | --- | --- | --- | --- | --- | --- |
| Lin et al [48] | Wireless three-lead ECG device. | Custom-built | ECG | 512 Hz | Every 6 seconds | Yes | Delivered to a smartphone or transmitted to a remote server for display and alerts. |
| Hu et al [47] | Wearable ECG sensor | iBoSen | ECG, EEG, respiration rate, skin temperature | - ECG: 100 Hz  - EEG: 100 Hz  - Respiration rate: 10 Hz  - Skin temperature: 1 H | — | No (on a smartphone) | Displayed on a smartphone for real-time visualization and notification. |
| Lin et al [33] | Single-lead ECG patch | Custom-built | ECG | 1200 Hz | Real-time | No (on the cloud) | Displayed on a smartphone and a web application. |
| Lin et al [36] | Wearable ECG patch. | Custom-built | ECG | — | — | No (on a smartphone) | Displayed on a smartphone. |
| Wasserlauf et al [45] | Smartwatch with ECG sensor | Apple Watch Series 2 paired with KardiaBand | Heart rate, activity level, ECG | Every 5 seconds | — | Yes | Notifications prompting ECG recordings. |
| Zhu et al [32] | Smartwatch and ECG patch. | Samsung Galaxy Watch Active 2, BioTel ePatch | PPG, acceleration, and ECG | PPG and acceleration 25 Hz, ECG continuous | Real-time | Yes | Displayed on a smartphone and in the cloud. |
| Fu et al [34] | Portable single-lead ECG device. | Custom-built | ECG | 125 Hz | Real-time | No (on the cloud) | Sent to a smartphone application for user display. |
| Ergen [38] | Aerogel-based nano-tattoo. | Custom-built | Electrical signals from skin-muscle interfaces. | — | — | — | — |
| Pramukantoro and Gofuku [30] | Chest strap | Polar H10 | Heart rate | 1 Hz | 1 Hz | No | Processed through real-time middleware on a local computer. |
| Nguyen et al [35] | PPG sensor patch | Custom-built | PPG | 500 Hz | — | Yes | — |
| Jenifer et al [37] | IoT-based wearable sensor module. | Custom-built | Heart rate, temperature, acceleration | Every 10 minutes | — | Yes | Displayed locally on an OLED screen. |
| Colombage et al [42] | Smartwatch | Mi Smart Band 6 | Blood pressure, heart rate, respiratory rate, body temperature, calories burned | Every hour | Real-time | No (on the cloud) | Delivered via smartphone and web applications for real-time monitoring and alerts. |
| Ye et al [31] | Wearable ECG sensors. | Custom-built | ECG | Event-driven based on amplitude thresholds | — | Yes | Displayed on the wearable device. |
| Howard et al [40] | Acoustic biosensors with ECG. | Cardiac Performance System | Cardiac acoustic signals and ECG | Continuous | — | Yes | Displayed on the Cardiac Performance System patient monitor. |
| Islam et al [41] | IoT-based sensors. | MAX30100 (pulse oximeter), AD8232 (ECG). MLX90614 (body temperature) | ECG, heart rate. SPO2, body temperature | ECG 250 Hz | Real-time | No (on the cloud) | Alerts and reports delivered via a smartphone application to patients and healthcare providers. |
| Mary et al [43] | Wearable sensors integrated into a Body Area Sensor Network | — | ECG | Continuous | Real-time | No (on the cloud) | Real-time data and alerts delivered to health monitoring personnel via a mobile device or computer interface. |
| Poh et al [44] | Study Watch (wrist-worn) | Verily | ECG, PPG | Every 15 minutes | — | Yes | Alerts for irregular pulse and ECG recording instructions delivered via watch vibrations and screen notifications. |
| Gavidia et al [46] | Smartwatches and fitness bands capable of measuring R-to-R intervals. | — | Heart rate | Every 15 seconds | — | — | — |
| Hannan et al [39] | IoT-based wearable system with multiple sensors. | Custom-built | ECG, Galvanic Skin Response, temperature sensor, SPO2 | — | Real-time | No (on the cloud) | Alerts delivered to caregivers and patients via an Android smartphone application. |
