# Supplementary material for: AI-Driven Real-Time Monitoring of Cardiovascular Conditions With Wearable Devices: Scoping Review
Source: JMIR Mhealth Uhealth. 2025 Nov 11;13:e73846. doi: 10.2196/73846 (PMC12777649; doi:10.2196/73846)
Supplement: Multimedia Appendix 6 [file mhealth_v13i1e73846_app6.docx]

Multimedia Appendix 6 (a). Characteristics of AI algorithms in the included studies.

| Reference | Outcome type | Output | Annotation | Features/Raw Data |
| --- | --- | --- | --- | --- |
| Lin et al [48] | Detection | Classes (Normal sinus rhythm, Atrial Fibrillation, Tachycardia, Bradycardia, Asystole, Wide QRS Complex) | Annotated by cardiologists based on 12-lead ECG interpretations | R-R intervals, QRS durations, RR intervals features |
| Hu et al [47] | Detection | Classes (Normal, Premature Ventricular Contraction, Atrial Premature Contraction, Invalid signal segment) | Used MIT-BIH | ECG, acceleration signals |
| Lin et al [33] | Detection | Classes (Normal, AF, Atrial Flutter, Ventricular Fibrillation) | Used MIT-BIH | ECG |
| Lin et al [36] | Detection | Classes (Normal, AF, Ventricular Tachycardia) | Used MIT-BIH and annotated by cardiologists | ECG features: RR intervals, QRS width, and signal morphology characteristics. |
| Wasserlauf et al [45] | Detection | Classes (AF or Non-AF) | — | ECG, heart rate, motion index derived from pedometer data |
| Zhu et al [32] | Detection | Classes (Normal, AF) | Annotated using clinical ground truth provided by BioTel ePatch ECG recordings | PPG features: signal quality, heart rate variability, pulse irregularity metrics; acceleration features: motion artifacts, activity levels |
| Fu et al [34] | Detection | Classes (AF, Ventricular Tachycardia, Bundle Branch Block, Normal) | Annotated by cardiologists | ECG |
| Ergen [38] | Detection | Classes (AF, Normal) | — | Skin-muscle interface electrical signals during motion. |
| Pramukantoro and Gofuku [30] | Detection | Classes (Normal, Supraventricular Ectopic Beat, Ventricular Ectopic Beat, Fusion Beat, Unknown) | Used the MIT-BIH public dataset | RR intervals and statistical features from RR intervals |
| Nguyen et al [35] | Detection | Classes (Normal, AF). | Annotated by cardiologists | PPG features: waveform shape, rhythm irregularities, and signal quality metrics |
| Jenifer et al [37] | Detection | Classes (Normal, Abnormal) | Used a public dataset from Kaggle | Pulse rate, body temperature, acceleration |
| Colombage et al [42] | Detection | Classes (heart failure: Yes/No; heart failure severity: Type II, Type III) | Used a public dataset | Resting blood pressure, maximum heart rate, body temperature, respiratory rate, calories burned, blood sugar levels, fasting blood sugar levels |
| Ye et al [31] | Detection | Classes (Normal, Abnormal) | Used MIT-BIH | ECG features: QRS complex localization, RR intervals, QRS durations, window accumulation features |
| Howard et al [40] | Detection | Continuous regression values (Left Ventricular Ejection Fraction expressed as a percentage) | — | Acoustic signals: Temporal, amplitude-based, and spectral features derived from heart sound signals ECG signals: Timing information for segmenting acoustic signals. |
| Islam et al [41] | Detection | Classes (Normal, Supraventricular Premature Beat, Premature Ventricular Contraction, Fusion of Ventricular and Normal Beat, Unclassifiable Beat) | Used MIT-BIH | ECG signals, and heart rate measurement. SPO2, body temperature |
| Mary et al [43] | Detection | Classes (Normal, Abnormal) | — | ECG signals collected via wearable sensors |
| Poh et al [44] | Detection | Classes (AF, Non-AF, Unanalyzable, Not enough data/off-wrist) | Annotated by cardiologists | PPG: Heart rate variability and inter-beat intervals. ECG: Single-lead waveform features |
| Gavidia et al [46] | Prediction | Classes (Sinus Rhythm, Pre-AF, AF) | Annotated by cardiologists | RR intervals derived from ECG data |
| Hannan et al [39] | Prediction | Classes (Less Critical, More Critical, and Normal Cardiac Condition) | — | ECG, galvanic skin response, body temperature, SPO2 |

Multimedia Appendix 6 (b). Characteristics of AI algorithms in the included studies.

| Reference | Features selection | Machine-/ deep-learning | Algorithms | Performance | inference frequency | inference time | Deployed on |
| --- | --- | --- | --- | --- | --- | --- | --- |
| Lin et al [48] | — | Machine | Expert rule-based System | - Accuracy: 0.94  - Positive Predictive Value: 0.99  - Sensitivity: 0.95  - False Negative Rate: 0.07  - True Negative Rate: 0.00 | Every 6 seconds | Less than 6 seconds | Wearable device |
| Hu et al [47] | — | Machine | Layered Hidden Markov Model | - Accuracy: 0.99  - Sensitivity: 0.98  - Positive Predictive Value: 0.97 | Less than 1 second | Less than 1 second | Smartphone |
| Lin et al [33] | — | Deep | CNN | - Accuracy: 0.95 | Real-time | Less than 5 seconds | Cloud |
| Lin et al [36] | — | Machine | Decision tree | - Accuracy: 0.97 | Real-time | — | Smartphone |
| Wasserlauf et al [45] | — | Deep | CNN | - Episode Sensitivity: 0.98  - Duration Sensitivity: 0.98  - Subject Sensitivity: 0.83  - Positive Predictive Value: 0.40 | Every 5-6 seconds | — | Wearable device |
| Zhu et al [32] | — | Machine | Logistic regression | - Sensitivity: 0.88  - Specificity: 0.97  - Pearson correlation for AF burden estimation: 0.98 | Every 5 minutes | Real-time | Wearable device |
| Fu et al [34] | — | Deep | CNN and recurrent neural network | - AUC-ROC: 0.98 | Real-time | — | Cloud |
| Ergen [38] | — | Deep | Neural network | — | — | — | — |
| Pramukantoro and Gofuku [30] | — | Machine | Decision tree, random forest, k-nearest neighbor, SVM, neural network | - Accuracy: 0.99  - Precision: 0.99  - Recall: 0.99  - F1-Score: 0.99 | 1 Hz | Less than 1 second | Local computer |
| Nguyen et al [35] | — | Deep | 1D and 2D CNN | - Accuracy: 0.98  - Sensitivity: 0.97  - Specificity: 0.99 | Real-time | — | Wearable device and cloud |
| Jenifer et al [37] | — | Machine | Decision tree | - Accuracy: 0.95 | Real-time | — | Wearable device |
| Colombage et al [42] | — | Machine | Logistic regression, random forest | - F1-Score: 0.72 | Real-time | — | Cloud |
| Ye et al [31] | — | Deep | Neural network | - Accuracy: 0.93 | 0.2 seconds | 0.2 seconds | Wearable device |
| Howard et al [40] | — | Deep | Neural network | - AUC-ROC: 0.97 | Real-time | — | Wearable device |
| Islam et al [41] | — | Deep | CNN with attention layers | - Macro average precision: 0.94  - Macro average recall: 0.84  - Macro average F1-score: 0.88  - Weighted average precision: 0.98  - Weighted average recall: 0.98  - Weighted average F1-score: 0.98  - Overall F1-score: 0.98  - Overall accuracy: 0.98 | Real-time | — | Cloud |
| Mary et al [43] | MLDA for dimensionality reduction | Deep | CNN | - Accuracy: 0.98 | Real-time | — | Cloud |
| Poh et al [44] | — | Deep | CNN | - Sensitivity: 0.96  - Specificity: 0.98  - Negative Predictive Value: 0.81  - Negative Predictive Value: 1.00  - AF Burden Correlation (R²): 0.99 | Every 15 minutes | — | Wearable device |
| Gavidia et al [46] | — | Deep | CNN | - Accuracy: 0.83  - F1-score: 0.85  - AUC-ROC: 0.90  - AUC-PR: 0.88 | Every 15 seconds | 100 milliseconds | — |
| Hannan et al [39] | — | Machine | Random forest, SVM, AdaBoost | - Accuracy: 0.98 | Real-time | 2 seconds | Cloud |
