# Supplementary material for: AI-Driven Real-Time Monitoring of Cardiovascular Conditions With Wearable Devices: Scoping Review
Source: JMIR Mhealth Uhealth. 2025 Nov 11;13:e73846. doi: 10.2196/73846 (PMC12777649; doi:10.2196/73846)
Supplement: Multimedia Appendix 7 [file mhealth_v13i1e73846_app7.docx]

Multimedia Appendix 7 (a). Robustness analysis of the AI algorithms in the included studies.

| Reference | Input perturbations and alterations | Missing data | Label noise |
| --- | --- | --- | --- |
| Lin et al [48] | Preprocessing uses band-pass and notch filters to eliminate noise and artifacts. | The QRS detection algorithm employs dynamic thresholding to detect missing beats. | Data were annotated by experienced cardiologists to maintain high-quality labels. |
| Hu et al [47] | The circuit minimizes noise from impedance and capacitance changes, with built-in motion artifact handling via accelerometer data. | — | Relied on high-quality labels from the MIT-BIH Arrhythmia Database. |
| Lin et al [33] | Noise reduction is achieved using an 8-point moving average filter, with baseline drift corrected through polynomial fitting. | — | Relied on high-quality annotations from the MIT-BIH dataset. |
| Lin et al [36] | Preprocessing utilizes low-pass and high-pass Butterworth filters along with notch filters to eliminate noise and artifacts. | — | Relied on high-quality annotations from the MIT-BIH dataset. |
| Wasserlauf et al [45] | Motion index calculation was incorporated into the model to account for motion artifacts. | The system managed missing data by including only continuous, time-synchronized inputs and discarding intervals lacking sufficient data for analysis. | Annotations were based on validated automated ECG classification algorithms to reduce uncertainty in training labels. |
| Zhu et al [32] | Preprocessing employs a bandpass filter to remove frequencies outside the heartbeat spectrum, a motion detection block for artifact reduction, and a sensor contact monitor to ensure signal quality. | — | The study utilized clinically validated annotations from ECG recordings reviewed by board-certified cardiologists and certified ECG technicians. |
| Fu et al [34] | Low-pass and high-pass filters remove noise and baseline drift during preprocessing, while a notch filter eliminates artifacts. | — | At least two cardiologists manually annotated and validated each label to ensure accuracy. |
| Ergen [38] | Graphene aerogel-based nano-tattoos and AI algorithms minimize noise and motion artifacts, ensuring reliable signal acquisition during movement. | — | — |
| Pramukantoro and Gofuku [30] | — | — | — |
| Nguyen et al [35] | A bandpass filter is used to denoise and eliminate motion artifacts from the PPG signal. | — | Cardiologists validated data annotations to ensure high-quality labels. |
| Jenifer et al [37] | — | — | — |
| Colombage et al [42] | Users were instructed to remain at rest during blood pressure measurements to minimize artifacts. | — | Publicly available datasets with labeled data from trusted sources were used for model training, reducing uncertainty in labels. |
| Ye et al [31] | An event-driven architecture manages variable sampling and minimizes noise. | — | Relied on high-quality annotations from the MIT-BIH Arrhythmia Database |
| Howard et al [40] | Acoustic signals undergo preprocessing with filtering techniques to reduce low- and high-frequency interference from motion, speech, or environmental noise. | — | Ground truth labels were performed by experienced echo cardiographers. |
| Islam et al [41] | ECG signals are preprocessed using a bandpass filter (0.5–100 Hz) for noise removal, resampled to 125 Hz, and normalized for consistent feature extraction. | — | Relied on high-quality annotations from the MIT-BIH dataset. |
| Mary et al [43] | ECG image preprocessing includes noise reduction, heartbeat alignment, and data standardization to minimize signal acquisition errors and measurement inconsistencies. | — | Labels were validated using a combination of manually extracted time-domain features and model-extracted features, ensuring consistency and accuracy. |
| Poh et al [44] | Unanalyzable signal periods due to motion and peripheral circulation were excluded. | The model excluded unanalyzable intervals and leveraged redundancy in continuous monitoring to ensure sufficient data for decision-making. | Certified cardio graphic technicians and electrophysiologist triple-reviewed annotations to minimize labeling errors during training. |
| Gavidia et al [46] | Preprocessing utilizes a band-pass filter and the Pan-Tompkins algorithm to reduce noise and improve signal quality. | — | Labels for AF were assigned by cardiologists to minimize errors. |
| Hannan et al [39] | Preprocessing includes data cleaning and anomaly removal to minimize the impact of noisy or inconsistent sensor data. | — | Biomarkers recorded from tertiary hospitals ensure reliability in measurement standards. |

Multimedia Appendix 7 (b). Robustness analysis of the AI algorithms in the included studies.

| Reference | Imbalanced data | Feature selection and extraction | Model specification and learning | External data and domain shift |
| --- | --- | --- | --- | --- |
| Lin et al [48] | — | Focused on R-R intervals, RRI variability, and QRS durations, robust to signal variations. | Threshold-based expert system with parameters tuned to handle variations in ECG patterns. | — |
| Hu et al [47] | Weighted metrics for evaluation; no specific balancing technique mentioned. | Used predefined ECG and accelerometer features to ensure consistency across physical states. | Incorporated a layered Hidden Markov Model for adaptive classification. | — |
| Lin et al [33] | — | — | Lightweight CNN with hyperparameter tuning based on experimental tests. | Validated with clinical trials from patients in a hospital environment as well as MIT-BIH database. |
| Lin et al [36] | — | Focused on R-R intervals, PR intervals, and P-wave duration, and P-peak location , robust to motion artifacts or other signal variations. | Optimization of decision tree depth using cross-validation to prevent overfitting while maintaining high classification accuracy | Updating decision tree coefficients based on user-specific ECG data uploaded to the cloud server, adapting to individual variations. System has been used in 40 human trials at time of publication. |
| Wasserlauf et al [45] | — | Features were extracted in fixed time intervals, ensuring robustness to noise and variability in input signals during preprocessing. Additionally, intervals with missing ECG data were discarded | The CNN architecture included dropout layers and batch normalization to ensure generalization and reduce overfitting during training. | Trained on data from 7500 users; validated on a separate cohort from Northwestern Memorial Hospital. No formal domain shift analysis, but good generalization across cohorts. |
| Zhu et al [32] | Decision thresholds were set with an emphasis on AF classification due to cohort’s prior history of AF diagnosis or at-risk status. | Focused on key PPG and accelerometer features like heart rate variability, inter-beat interval, and motion compensation metrics to ensure robustness. | Decision thresholds validated on prior development datasets. | Algorithms were validated on diverse cohorts prior to large-scale study, including healthy participants and AF patients in free-living and clinical settings. |
| Fu et al [34] | — | ECG measurements done using QRS complex detection and evaluated using case studies. | Combined CNN and recurrent neural network layers optimized for time-series and spatial feature learning. | — |
| Ergen [38] | — | — | Neural networks optimized for motion-resistant PPG signal analysis. | — |
| Pramukantoro and Gofuku [30] | Addressed using oversampling techniques, such as SMOTE | Used predefined low-complexity RR interval features. | Evaluated multiple algorithms to optimize for simplicity and real-time efficiency | — |
| Nguyen et al [35] | — | 1D-CNN is trained using expert labelled PPG data and then used for signal quality assessment to ensure features are only extracted from reliable PPG signals. | Combined CNN models (1D-CNN for quality assessment and 2D-CNN for detection) optimized for real-time and cloud-based processing. | — |
| Jenifer et al [37] | — | Direct use of sensor-derived features (pulse rate, temperature, and accelerometer data) for consistent input variability management. | The decision tree classifier was optimized for lightweight computation on an edge device. | — |
| Colombage et al [42] | SMOTE was applied to balance classes in the datasets during training. | Features were selected based on their collectability through the smartwatch or user inputs to reflect wearable conditions. | — | — |
| Ye et al [31] | Multi-cycle heartbeat reconstruction to reduce the imbalance between normal and abnormal beats | Multi-cycle heartbeat segment reconstruction to preserve RR features and QRS features and reduce variability. | Utilized a simplified neural network structure optimized for low-power hardware with fixed-point quantization but no mention of hyperparameter selection and tuning | — |
| Howard et al [40] | — | Features were extracted from acoustic and ECG signals based on their strong correlation with LVEF, ensuring reliability across conditions. | The model was locked and pre-trained with a predefined regression-based neural network architecture to prevent variability during deployment and ensure consistent performance. | The platform was tested on a diverse population with a wide range of LVEF values, various cardiac conditions, and belonging to 3 different clinical settings to ensure generalizability. |
| Islam et al [41] | — | The attention layer in the CNN focuses on the most informative signal regions, improving robustness to variability in feature extraction. | The model parameters, such as filter sizes, dropout rates, and learning rates, were fine-tuned using grid search to improve consistency and performance across different data inputs. | — |
| Mary et al [43] | — | MLDA was employed to extract and prioritize the most essential features, reducing the impact of variability and irrelevant features. | An adaptive deep neural network architecture was designed with multiple nonlinear layers to improve robustness and increase the model’s capacity to generalize across data. | — |
| Poh et al [44] | — | — | — | The model was evaluated across diverse populations, activity levels, and Fitzpatrick skin tone categories to address domain shift. |
| Gavidia et al [46] | — | Simplification to R-to-R intervals, extracted directly from ECG data, to ensure consistency and robustness across diverse wearable devices. | Use of cross-validation during CNN model training to tune optimal threshold and moving average for tradeoff between sensitivity and specificity. | Validation on external datasets from Argentina and France to assess generalizability across different populations and care settings |
| Hannan et al [39] | — | Use of domain-specific features (e.g., ECG, SPO2, GSR, pulse rate) to improve feature reliability and relevance. | Comparison of multiple machine learning algorithms (Random Forest, SVM, AdaBoost) to select the best-performing model (Random Forest) based on accuracy and error rate. | — |
